# Infantile allergic diseases: a cohort study prenatal fish intake and mercury exposure context

**DOI:** 10.1186/s12889-024-18008-9

**Published:** 2024-02-22

**Authors:** Surabhi Shah, Hae Soon Kim, Yun-Chul Hong, Hyesook Park, Mina Ha, Yangho Kim, Ji Hyen Lee, Eun-Hee Ha

**Affiliations:** 1 Department of Environmental Medicine, Ewha Womans University College of Medicine, 808-1, Magok-dong, Gangseo-gu, 07804 Seoul, Republic of Korea; 2 Department of Pediatrics, Ewha Womans University College of Medicine, 808-1, Magok-dong, Gangseo-gu, 07804 Seoul, Republic of Korea; 3 Department of Preventive Medicine, College of Medicine, Seoul National University, Seoul, Republic of Korea; 4 Department of Preventive Medicine, College of Medicine, Ewha Womans University, Seoul, Republic of Korea; 5 Department of Preventive Medicine, Dankook University College of Medicine, Cheonan, Republic of Korea; 6 Department of Occupational and Environmental Medicine, University of Ulsan College of Medicine, Ulsan University Hospital, Ulsan, Republic of Korea; 7 System Health & Engineering Major in Graduate School (BK21 Plus Program), Ewha Womans University, Seoul, Republic of Korea

**Keywords:** Prenatal fish intake, Allergic diseases, Mercury

## Abstract

**Background:**

Allergic diseases (ADs) have been increasingly reported in infants and children over the last decade. Diet, especially the inclusion of fish intake, may help to lower the risk of ADs. However, fish also, can bioaccumulate environmental contaminants such as mercury. Hence, our study aims to determine what effects the type and frequency of fish intake have on ADs in six-month-old infants, independently and jointly with mercury exposure.

**Methods:**

This study is part of the prospective birth cohort: Mothers and Children’s Environmental Health (MOCEH) study in South Korea. Data was collected on prenatal fish intake, prenatal mercury concentration and ADs for infants aged six months for 590 eligible mother-infant pairs. Logistic regression analysis was conducted to evaluate the risk of prenatal fish intake and mercury concentration on ADs in infants. Finally, interaction between fish intake and mercury concentration affecting ADs in infants was evaluated. Hazard ratios of prenatal fish intake on ADs in 6 month old infants were calculated by prenatal mercury exposure.

**Results:**

Logistic regression analysis showed that white fish (OR: 0.53; 95% CI 0.30–0.94; *P* < 0.05) intake frequency, once a week significantly decreased the risk of ADs in infants. Stratification analysis showed that consuming white fish once a week significantly reduced the hazard of ADs (HR: 0.44; 95% CI 0.21–0.92; *P* < 0.05) in infants in the high-mercury (≥ 50th percentile) exposure group.

**Conclusion:**

The result indicates that prenatal white fish intake at least once a week reduces the risk of ADs in infants, especially in the group with high prenatal mercury exposure.

**Supplementary Information:**

The online version contains supplementary material available at 10.1186/s12889-024-18008-9.

## Background

Allergic diseases (ADs) in children are a major public health concern. In particular, ADs begin during infancy and are common in children [1]. The prevalence of allergic diseases in South Korean children with asthma, allergic rhinitis and atopic dermatitis was 0.9%, 9.0%, and 5.9%, respectively [2]. A systematic review showed an increasing trend of allergic conjunctivitis, atopic dermatitis, and food allergies in children in South Korea [3]. Environmental exposure by a pregnant women’s diet is considered as a factor in the development of ADs and further ‘atopic march’ in children [4]. Although research suggests that dietary changes may also contribute to these allergic reactions [5]. A Mediterranean diet that includes fish is recommended as a healthy dietary pattern compared to a Western diet [6]. The beneficial nutrients in fish intake have garnered attention for the primary prevention of allergic diseases. A study representing a national sample of South Korean women showed that daily fish consumption ranged from 18.65 to 26.44 g/day [7], which is comparatively higher than their American counterparts [8].

Fish is rich in n-3 polyunsaturated fatty acids (PUFAs), such as docosahexaenoic acid (DHA) and eicosapentaenoic acid (EPA), which produce anti-inflammatory effects that may lower the risk of allergic diseases [9]. The essential amino acids in fish, including vitamins A, D, and B_12_, and selenium, might help to combat the development of allergic diseases [10]. However, fish also can bioaccumulate environmental contaminants, such as mercury, polychlorinated biphenyls, perfluorinated chemicals, dioxins, and lead, which can increase the risk of allergies [11–13]. A study in the United States found that fish consumption is associated with an increase in mercury concentrations in women of reproductive ages [14]. Another study in South Korea found that blood mercury concentration was associated with fish consumption [15]. Most countries recommend that large predatory fish such as tuna, swordfish, king mackerel, tilefish, and shark should not be consumed during pregnancy due to their high levels of mercury.

The extent to which fish intake during pregnancy protects against allergic symptoms during childhood remains unclear. Two systematic reviews were conducted on the role of fish intake in preventing allergic diseases, which found that most epidemiological studies concluded that fish intake during pregnancy provided a protective effect [16, 17]. However, another systematic review found inconsistent results for prenatal fish intake relating to the incidence of atopic dermatitis in infants [18]. In the study of cell in vitro, marine n-3 fatty acids ameliorated mercury toxicity by decreasing apoptosis or by reducing mercury uptake [19]. Nevertheless, until recently, the net effect of the beneficial nutrients and harmful contaminants contained within fish and associated with ADs has been under-studied and remained unclear.

In South Korea, women of reproductive ages consume larger quantities of fish and seafood [7]. Following an increase in the incidence of allergic diseases in infants [2], the guidelines on the recommended types and amounts of fish to consume to reduce the incidence of ADs in offspring remain improper. Thus, in the present study, we evaluated the effects of the frequency of maternal intake of white fish, blue fish, and shellfish and the exposure to mercury during pregnancy on the incidence of ADs in six-month-old infants in South Korea. Further, we explored the interaction between fish intake and mercury and their effects on ADs in infants.

## Methods

### Study participants

**Fig. 1:**
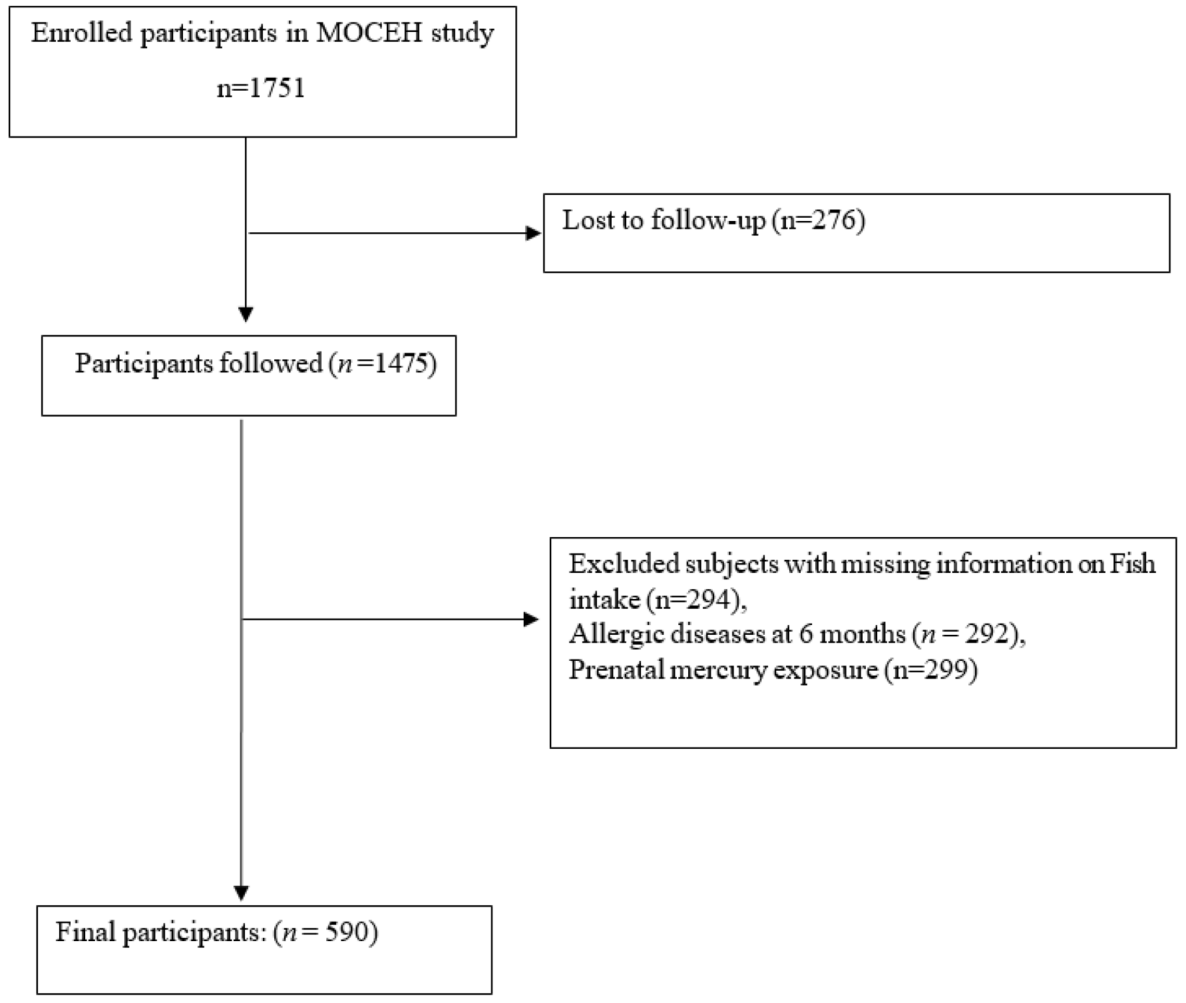
Selection of study participants

This study is part of the Mothers and Children’s Environmental Health (MOCEH) study. This prospective birth cohort study examined the effect of environmental pollutants on children’s growth, development, and disease status. The details of the MOCEH study have been described previously [20]. The MOCEH study enrolled 1,751 pregnant women over the age of 18 years and in the early period of pregnancy (less than 20 weeks of gestational age) from 2006 to 2010. In our study, we excluded women who did not participate in the follow-up (*n* = 276). Further, we selected 590 mother–children pairs for whom information on prenatal fish intake, prenatal mercury concentration, and allergic diseases was available for infants at six months (Fig. 1). Sensitivity analysis between eligible participants and those excluded showed that there was no significant difference in the characteristics of participants, as demonstrated in supplementary table 1.

Written informed consent was obtained from the pregnant women for themselves and on behalf of their children. The study protocol was approved by the Institutional Review Board of Ewha Womans University Hospital, Dankook University Hospital, and Ulsan University Hospital. Upon enrollment, a detailed questionnaire was used to obtain information from the participants on their demographics, socioeconomic factors, residential characteristics, medical and reproductive history, exposure to occupational hazards, alcohol consumption, and nutritional habits.

### Assessment of maternal fish intake

Enrolled pregnant women were asked to complete a food frequency questionnaire (FFQ) during their prenatal visits to assess their fish intake. The FFQ method has been previously validated [21]. However, the version used in the present study only contained 107 of the original 113 items due to the removal of alcoholic beverages because detailed alcohol-drinking behaviors were determined separately in our study. The FFQ consisted of a list of foods on selection of 9 frequency categories: 3 times daily, twice daily, once daily, 5 or 6 times weekly, 3 or 4 times weekly, once or twice weekly, 2 or 3 times monthly, once monthly, and never or seldom. Maternal consumption of white fish (Pollock, hairtail, and yellow croaker), blue fish (mackerel, beak, and Spanish mackerel), and shellfish (shrimp and crab) were evaluated. Detailed information was obtained on the frequency of consuming boiled, fried, or grilled fish servings.

The participants’ dietary intakes for nutrients and food groups were assessed using a computerized nutrient-intake assessment software program (CAN-Pro 3.0, Korean Nutrition Society, Seoul, Korea). Information regarding the intake of n-3 fatty acids supplements and the frequency of consumption was collected from the participants who took dietary supplements. However, our study participants did not consume dietary supplements containing n-3 fatty acids.

### Assessment of prenatal mercury exposure

Maternal blood samples were collected during early pregnancy (< 20 gestational weeks) and late pregnancy (> 28 gestational weeks). Cord blood samples were collected at the time of birth. The blood samples were stored at − 70 °C until analysis. The mercury (Hg) analysis was performed by atomic absorption spectrometry (DMA-80; Milestone, Bergamo, Italy). The samples were dried in an oxygen stream passed through a quartz tube located inside a controlled heating coil. The combustion gasses were decomposed in a catalytic column at 750 °C. The Hg vapor was collected on a gold amalgamation trap and further desorbed for quantification. The Hg analyses were performed using standardized quality-control procedures. Internal quality control was used for each series of analyses. The precision and accuracy of the Hg measurements were periodically verified by an external quality control program (interlaboratory calibration exercises). The limit of detection (LOD) was 0.158 µg/L, while no sample was below the LOD.

### Assessment of allergic diseases

Here, the ADs were defined as at least one of the following: atopic dermatitis, asthma, milk allergies, and any other allergic diseases. Mothers/caregivers were asked about infantile symptoms, medical history, and the diagnosis of ADs through a questionnaire when the infants were 6 months old [22]. The ADs were considered present if the mother/caregiver answered yes to the questions: “Did a doctor ever diagnose your child with ADs?” or “Has your child ever visited the hospital for ADs treatment?”

### Statistical analysis

Descriptive analyses of the mothers and their infants in our birth cohort at six months were determined using the *t*-test for continuous variables and the chi-square test for categorical variables. After descriptive analyses, the prenatal mercury concentrations were natural log-transformed because of their skewed distribution. The geometric means (GMs) and percentiles of the prenatal mercury concentrations were calculated according to the fish intake frequency.

A logistic regression analysis was performed to estimate the odds ratio (OR) and 95% confidence intervals (95% C.I. ) to assess the relationship between mother’s fish intake (type and frequency) and ADs in their infants at six months. These models were adjusted for mother and infant factors, including the infant’s gender, maternal age (< 30 and ≥ 30 years), pre-pregnancy body mass index (BMI: < 18.5, 18.5–22.9, ≥ 23 kg/m^2^), maternal educational level (< 12 or ≥ 12 years of schooling), parity (0 or 1), parenteral history of allergy (yes or no), secondhand smoke exposure (< median, ≥ median exposure), colostrum feeding (yes or no), breastfeeding (yes or no), and gestational age (weeks). Due to the limited sample size, the missing variables for the mothers’ age, education, pre-pregnancy BMI, and parity were converted into an unknown category and included in the analyses. The full distribution of these characteristics for the mothers is shown in supplementary table 2. A multiple-model logistic regression analysis was performed, which included both the fish intake and mercury level of mothers in a single model. The interaction of prenatal mercury exposure and fish intake of mothers affecting ADs in their six months old infants was calculated on the multiplicative scale by adding multiplication terms between mercury and fish intake in the logistic regression model [23, 24]. Next, Cox proportional hazard model was used for stratification analysis according to the 50th percentile exposure of prenatal mercury concentration in mothers, to estimate the hazard ratio (HR) and 95% C.I.

Statistical significance was defined as *p-*value < 0.05. Data were analyzed using SAS statistical software (version 9.4; SAS Institute Inc., Cary, NC, USA).

## Results

The mean age of the mother was 30 years and 75% had completed high school. Almost 50% were prime parous with a mean gestational age of 39 weeks. The average pre-pregnancy BMI was 21.8 kg/m^2^, while 31% of mothers and 26% of fathers had a history of allergic disease. The mean birth weight of their newborns was 3.27 kg, and 48% of the babies were girls (Table 1). Overall, 316 (54%) mothers reported eating fish more than once per week, while 274 (46%) reported eating fish less than once a week. Almost 88% of the mothers reported consuming white and blue fish less than once a week, while 93% reported eating shellfish less than once a week (Table 1).

**Table 1:** General characteristics of the study participants

*Mother’s*		Total (*n* = 590)	Infant’s Allergic diseases: no (*n* = 395)	Infant’s Allergic diseases: yes (*n* = 195)
Age (years)		30.2 ± 3.55	30.3 ± 3.63	29.9 ± 3.38
	≤ 30	349[59]	225[57]	124[64]
	> 30	241[41]	170[43]	71[36]
Educational level (years)*	≤ 12	148[25]	119[30]	29[15]
	> 12	436[75]	273[70]	163[85]
Family Income (million KRW/month)	< = 2	153[26]	103[26]	50[26]
	2–6	319[54]	212[54]	107[55]
	> 6	114[20]	78[20]	36[19]
Parity*	1	298[55]	183[51]	115[64]
	> 1	242[45]	178[49]	64[36]
Pre-pregnancy BMI		21.83 ± 3.35	21.96 ± 3.29	21.59 ± 3.45
(kg/m^2^)	< 18.5	55[12]	36[11]	19[12]
	18.5–22.9	276[59]	180[57]	96[62]
	≥ 23	138[29]	98[31]	40[26]
Total fish intake during pregnancy	< 1	274[46]	178[45]	96[49]
(times/week)	≥ 1	316 [54]	217[55]	99[51]
White fish intake during pregnancy*	< 1	501[85]	326[82]	175[90]
(times/week)	≥ 1	89[15]	69[18]	20[10]
Blue fish intake during pregnancy	< 1	523[88]	346[87]	177[91]
(times/week)	≥ 1	67[12]	49[13]	18[9]
Shell fish intake during pregnancy	< 1	552[93]	369[93]	183[93]
(times/week)	≥ 1	38[7]	26[7]	12[7]
Mother’s allergy history	No	402[69]	282[71]	122[63]
	Yes	186[31]	113[29]	73[37]
Father’s allergy history	No	439[74]	306[77]	133[68]
	Yes	151[26]	89[23]	62[32]
***Child’s***				
Gestational age (weeks)		39 ± 1.34	39 ± 1.42	39 ± 1.16
Birth weight (kg)*		3.27 ± 0.40	3.24 ± 0.40	3.31 ± 0.40
Infant’s gender*	Boy	307[52]	194[49]	113[58]
	Girl	283[48]	201[51]	82[42]

Mean ± SD, N [%]; *p-value < 0.05

Numbers do not always be the same total due to missing values

Of the infants with atopic dermatitis, 58% were boys and almost 64% were the firstborn child. Mothers of almost 90% of the infants with atopy consumed white, blue, and shellfish less than once a week. Nearly 37% of the mothers and 32% of the fathers of the infants with atopy had a history of allergy (Table 1).

**Fig. 2:**
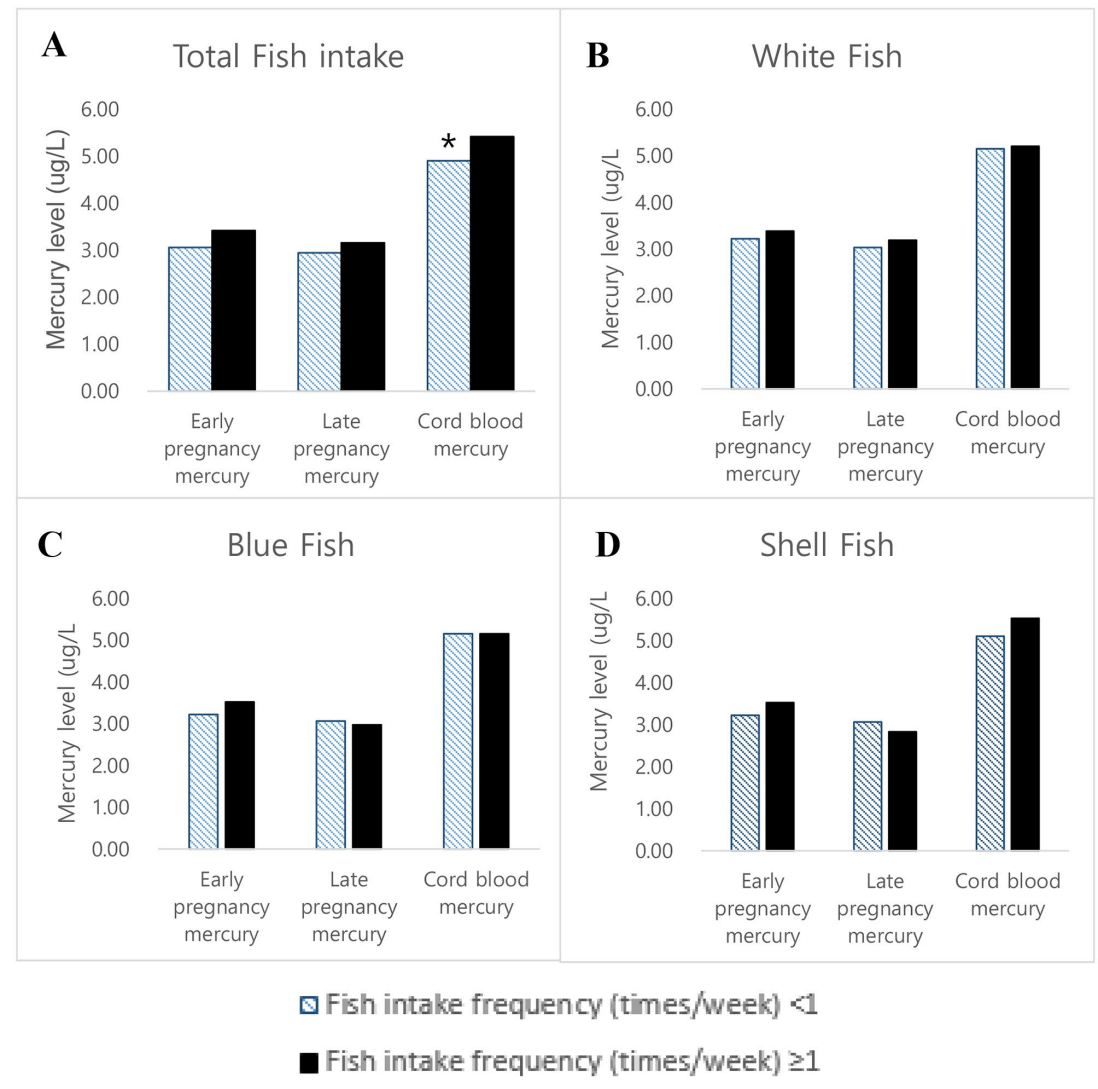
Prenatal mercury distribution according to type and frequency of fish intake

The GM of prenatal mercury exposure of mothers was 3.25, 3.09, and 5.20 µg/L at early pregnancy, late pregnancy, and in the cord blood, respectively (Table 2). The cord blood mercury concentration was significantly higher when the consumption of the total fish intake was higher than once per week (Fig. 2).

**Table 2:** Distribution of prenatal mercury concentration

Mercury level (ug/L)	GM ± GSD	Minimum	25th percentile	Median	75th percentile	Maximum
Early Pregnancy	3.25 ± 1.58	0.24	2.45	3.33	4.43	14.63
Late Pregnancy	3.09 ± 1.69	0.45	2.23	3.09	4.08	20.77
Cord Blood	5.20 ± 1.63	0.20	3.91	5.23	7.00	54.65

GM: Geometric mean and GSD: geometric standard deviation

Logistic regression analysis in a single model, which considered only mother’s fish intake showed that mother’s intake of white fish during pregnancy had a lower risk of allergic diseases (OR: 0.53; 95% CI 0.30–0.94) in their infants, a single model considering only mercury exposure in mothers showed an increased risk of allergic diseases in their infants; however, it did not reach statistical significance (Table 3). Multiple model analyses by mutually adjusting mother’s prenatal fish intake and mercury concentration showed that infants whose mothers consumed white fish more than once a week had a reduced risk of allergic diseases (Table 4). The multiplicative interaction between mothers white fish intake and cord blood mercury concentration was significant, indicating an effect modification of prenatal fish intake according to mercury exposure (Table 4). Thus, stratification analysis by prenatal mercury exposure was performed. Stratification analysis showed that mother’s consuming white fish more than once per week had a significant decrease in hazard of ADs (HR: 0.44; 95% CI 0.21–0.92) in their six-month-old infants in the high cord blood mercury (≥ 50th percentile) exposure group, compared to the low-cord blood mercury exposure group (Table 5). The results of the stratification analysis from the early and late pregnancy mercury concentration on the risk of ADs in infants were not significant (Table 5).

**Table 3:** Effect of type and frequency of prenatal fish intake and prenatal mercury exposure on allergic disease in six-month-old infants in single model

Mother’s Fish intake During pregnancy	Infants Allergic diseases Unadjusted OR (95% C.I.)	Infants Allergic diseases Adjusted† OR (95% C.I.)
**Frequency ≥ 1 (times/week)**		
Total Fish^**¶**^	0.84 (0.60,1.19)	0.78 (0.53,1.14)
White fish^**¶**^	0.54 (0.31,0.91)*	0.53 (0.30,0.94)*
Blue fish^**¶**^	0.71 (0.40,1.27)	0.73 (0.39,1.36)
Shell fish^**¶**^	0.93 (0.46,1.88)	0.77 (0.34,1.73)
**Prenatal mercury concentration**		
Early Pregnancy^**★**^	1.33 (0.91,1.94)	1.55 (0.95,2.54)
Late Pregnancy^**★**^	0.96 (0.70,1.32)	1.05 (0.71,1.55)
Cord Blood^**★**^	0.96 (0.67,1.36)	1.21 (0.72,1.75)

Odds ratio (OR) and 95% confidence intervals (C.I.) estimated using logistic regression model

†Adjusted for mother’s age, education, parity, pre-pregnancy BMI, parental allergy history, gender of the child, secondhand-smoke exposure, colostrum and breastfeeding

*p-value < 0.05; ^**¶**^ Single model for fish intake without mercury concentration in one model; ^**★**^ Single model for prenatal mercury concentration without fish intake in one model

**Table 4:** Effect of type and frequency of prenatal fish intake and prenatal mercury exposure on allergic disease in six-month-old infant in multiple model

Allergic diseases in infants							
Mother’s Fish intake frequency in pregnancy (times/week)		Multiple model with early pregnancy mercury**	*p*-value for interaction^¶^	Multiple model with late pregnancy mercury**	*p*-value for interaction^¶^	Multiple model with cord blood mercury**	*p*-value for interaction^¶^
		OR (95% C.I.) †		OR (95% C.I.) †		OR (95% C.I.) †	
All type of fish	< 1	1.00 (ref.)		1.00 (ref.)		1.00 (ref.)	
Total Fish	≥ 1	0.76 (0.51,1.12)	0.99	0.79 (0.53,1.16)	0.93	0.79 (0.53,1.16)	0.12
White fish	≥ 1	0.52 (0.29,0.92)*	0.63	0.54 (0.30,0.95)*	0.57	0.54 (0.31,0.93)*	0.03*
Blue fish	≥ 1	0.70 (0.38,1.31)	0.71	0.73 (0.40,1.36)	0.86	0.74 (0.40,1.37)	0.17
Shell fish	≥ 1	0.76 (0.34,1.70)	0.21	0.76 (0.34,1.71)	0.78	0.78 (0.35,1.76)	0.46

Odds ratio (OR) and 95% confidence intervals (C.I.) estimated using logistic regression model

† Adjusted for mother’s age, education, parity, pre-pregnancy BMI, parental allergy history, gender of the child, secondhand-smoke exposure, colostrum and breastfeeding

*p-value < 0.05; ** Multiple model include both: fish intake and mercury level in one model. ^¶^ Multiplicative interaction between prenatal fish intake and mercury concentration

**Table 5:** Stratification analysis of type and frequency of fish intake during pregnancy on allergic diseases in six-month-old infants by prenatal mercury exposure

Fish intake frequency (times/week)		Early pregnancy Mercury		Late pregnancy Mercury		Cord blood mercury	
		< 50th percentile (*n* = 294)	≥ 50th percentile (*n* = 296)	< 50th percentile (*n* = 295)	≥ 50th percentile(*n* = 295)	< 50th percentile (*n* = 294)	≥ 50th percentile (*n* = 296)
		HR (95% C.I.)	HR (95% C.I.)	HR (95% C.I.)	HR (95% C.I.)	HR (95% C.I.)	HR (95% C.I.)
All type of fish	< 1	1.00 (ref.)	1.00 (ref.)	1.00 (ref.)	1.00 (ref.)	1.00 (ref.)	1.00 (ref.)
Total fish	≥ 1	0.86 (0.70,1.82)	0.93 (0.60,1.42)	0.82 (0.78,1.94)	0.95 (0.56,1.45)	0.75 (0.81,1.95)	0.83 (0.51,1.34)
White fish	≥ 1	0.68 (0.32,1.45)	0.57 (0.30,1.09)	0.73 (0.33,1.60)	0.64 (0.34,1.23)	0.62 (0.55,2.17)	0.44 (0.21,0.92)*
Blue fish	≥ 1	0.76 (0.59,2.63)	0.45 (0.21,0.96)	0.95 (0.49,2.25)	1.02 (0.26,1.22)	0.89 (0.47,2.15)	0.58 (0.28,1.23)
Shell fish	≥ 1	0.97 (0.62,3.65)	0.50 (0.18,1.41)	0.89 (0.30,2.40)	1.10 (0.30,1.91)	0.62 (0.19,2.05)	0.99 (0.43,2.27)

Hazard ratio (HR) and 95% confidence intervals (C.I.) was estimated using Cox regression model adjusted for mother’s age, education, parity, pre-pregnancy BMI, parental allergy history, gender of the child, secondhand-smoke exposure, colostrum and breastfeeding

*p-value < 0.05

## Discussion

The present study showed that white fish consumption by a pregnant woman was associated with a lower risk of ADs in their six-month-old infants. Stratification analysis by Hg concentration showed that prenatal fish intake at least once a week, especially of white fish, reduced the risk of ADs in infants at six months, even during high (≥ 50th percentile) prenatal mercury exposure.

A study conducted in Spain evaluated that increased frequency of fish consumption during pregnancy reduced the risk for allergic diseases in children [25]. Another study by Jedrychowski et al. found that prenatal fish intake had a protective effect against infantile eczema [26]. However, Pele and colleagues did not find an association between prenatal fish intake and allergic diseases in infants [27]. A systematic review of prenatal fish consumption and allergic diseases in their offspring could not confirm or deny the effect of maternal fish intake and allergic diseases in children due to inconsistent results [17]. The inconsistency in results in previous studies could be due to variation in study population, exposure assessment, differences in types and frequency of fish intake and infants and mother’s dietary pattern [28, 29]. Our study found a protective effect of consuming fish during pregnancy against ADs in infants at 6 months.

Prenatal mercury exposure can lead to the development of allergic diseases in infants and children [30]. Some studies showed that prenatal mercury exposure elevated the Immunoglobulin G (IgG) levels causing mercury-induced immunotoxicity and allergic diseases in children [31, 32]. While the exact mechanism for the association of Hg to ADs remains unclear, it is believed that it can modulate changes in both the humoral and cellular immunity biomarkers. Mercury exposure causes mitochondrial dysfunction and T cell apoptosis and increases Immunoglobulin E (IgE) levels, thereby increasing inflammation [33].

Fish forms a major part of the Korean diet [34], accounting for 20% of the energy consumed from animal sources [35]. The consumption pattern of white fish in our study was similar to another study of the representative national population [36]. Our study showed that around 85% of pregnant women reported a white fish intake of less than once a week, while more than 50% of pregnant women had a total fish intake of more than once a week. Although fish intake is a major contributing factor to the total mercury intake in the Korean population. But, other foods such as grains, especially rice grown in contaminated fields can also contribute to the total mercury intake [37]. However, according to previous research, mercury concentration in rice is relatively low in Korea, and there has been no significant association between grain consumption and blood mercury concentration [38]. Even though mercury exposure, fish intake has several health benefits like modulating cell growth, differentiation and neurodevelopment owing to PUFAs. But, higher fish consumption may lead to contradictory outcomes due to an increase in blood mercury levels. No previous study has reported on the relationship between prenatal fish intake, mercury levels, and ADs in infants.

Fish is a rich source of PUFAs that include EPA and DHA, which are important for healthy fetal development. PUFAs maintain protein function and membrane fluidity in cells and modulate the immune response by affecting the production of inflammatory cytokines and influencing the T helper 1 versus T helper 2 (Th2) balance [26]. Dietary PUFA intake during pregnancy has an immunomodulatory response that is associated with allergic diseases in offspring. A study by Dunstan et al. demonstrated that supplementation of PUFAs during pregnancy decreased the expression of Th2 cytokines in neonatal cells [39] and neonatal lipid peroxidation [40]. Another study showed that PUFAs were associated with a decrease in serum IgE levels, thereby reducing the immune response to allergen exposures [41]. Therefore, frequent fish consumption can have a protective effect against the negative actions of mercury on the immune system and the development of allergic diseases in infants. We found a significant interaction between fish intake and mercury concentration on a multiplicative scale. This indicates that in the high-mercury exposure group, an increased frequency of white fish intake reduced the odds of allergic diseases in infants six months of age.

The window of exposure is important in the development of immunological diseases, whereby greater immunomodulatory benefits of PUFAs are seen during early immune development [24]. As allergic responses are observed at birth and manifested in the first month of life, immune development likely begins at a very early age [42]. Another study showed attenuated immune responses in neonates whose mothers took fish oil supplements during pregnancy [39]. Thus, the results of our study shows, that more frequency of fish intake was associated with reduced risk of allergic diseases in infants.

However, fish intake is a source of mercury exposure. The cord blood mercury concentration in our study was lower than that previously reported in Japan, whereas it was substantially higher than the concentrations reported in the US and Canada [43]. Thus, Korean population has high blood mercury levels compared to Western countries. Seafood, especially fish and shellfish, contain lipid-soluble organic mercury that can easily be transported across cellular membranes [44]. Therefore, the mercury transports to the fetus crossing the blood-brain barrier in pregnant women consuming shellfish or fish with high amounts of organic mercury [45]. Mercury is associated with the development of ADs, as it has the ability to modulate the immune system through the proliferation of lymphocytes [30].

Generally, white fishes contain less fat, thereby a low amount of organic mercury. Moreover, the fat content in fish does not directly correlate with their PUFAs concentration. Thus, many fish species, e.g., white fishes, which are low in fat are also good sources of PUFAs [46]. Additionally, marine n-3 fatty acids may reduce mercury toxicity by decreasing apoptosis and reducing mercury uptake [19]. EPA may ameliorate methylmercury (MeHg) toxicity through its effects on Ca2 + homeostasis [19]. In addition, in mice pups fed diets spiked with DHA, a decreased accumulation of MeHg was observed in the brain [47]. In vitro studies using both neuronal cell lines and mice primary neuronal cells have also shown that DHA can decrease the uptake of MeHg [48, 49]. Commonly, the amount of mercury and PUFAs present in fish species are not consistently associated. Hence, different types of white fish, which are high in PUFAs and have low Hg concentrations, can be chosen for their health benefits and reduced Hg toxicity [46]. Therefore, a proper balance of health benefits could be achieved.

To our knowledge, this is the first birth cohort study to assess the effect of the type and frequency of dietary fish intake in pregnant women on ADs in infants, with regard to prenatal Hg exposure. Our study provides a recommendation for an important public health goal for pregnant women to incorporate fish in their diet, while also considering minimal mercury exposure and maximum nutritional benefits. The birth cohort study design allows for the proper examination of risk factors in early-life diseases. Another strength of our study is that we studied prenatal fish intake, which is important as prenatal fish intake may play a critical role in the early development of the immunological system. But, along with maternal fish intake various nutrients and factors like EPA and DHA, composition of breastmilk, trans-fatty acids, vitamin C, magnesium and calcium may affect the development of immune system and further the development of allergic diseases in children. Atopic dermatitis is also influenced by genetic factors [50–52]. In the group with low prenatal mercury exposure, the influence of unmeasured confounding factors, such as genetic immunity and interactions related to breast milk nutrition and other nutrients, may be more pronounced compared to the high prenatal mercury exposure group. Thus, we found a non-significant association between fish intake and ADs in the low mercury exposure group.

However, our study had some limitations: First, the diagnosis of the ADs was based on parental reporting, therefore, it could have been a subject of recall bias. However, it is unlikely that the parents would make an erroneous report of a diagnosis of allergic diseases. Nevertheless, the observed prevalence of allergic diseases is in accordance with previous reports performed in Korea. Skin prick tests or biochemical tests could not be performed given the age of the infants involved in this study (six months). Second, we have used frequency of fish intake, it may have biased our results, as quantity of fish intake could not be considered. Third, in the Cox proportional hazard model analysis, we could not get the exact time for fish intake, since the fish intake information was obtained from questionnaire during the participant's prenatal visit. Also, the results cannot be generalized because all the participants were Korean. Fourth, in this study, we did not consider other seafood’s like eel, salmon as they can be a possible source of considerable mercury exposure. We did not have any information on the organic or inorganic type of mercury exposure. Lastly, we adjusted for all potential confounders, such as parenteral allergy history and maternal pre-pregnancy BMI, although the exposure to other pollutants may have biased the results if they correlated with the type and frequency of fish intake and allergic diseases in children.

## Conclusion

The present study suggests that prenatal white fish intake reduces the risk of ADs in 6-month-old infants. In the event of high prenatal Hg exposure, white fish intake during pregnancy decreases the risk of ADs in their infants. Further, large-scale epidemiological studies are needed to corroborate these results, determine the strength of the association, and investigate the potential health effects later in life.

### Electronic supplementary material

Below is the link to the electronic supplementary material.

Supplementary Material 1

## Data Availability

The datasets generated and/or analyzed during the current study are not publicly available due to them containing information that could compromise research participant privacy but are available from the corresponding author on reasonable request.
