# Supplementary material for: Infantile allergic diseases: a cohort study prenatal fish intake and mercury exposure context
Source: BMC Public Health. 2024 Feb 22;24:568. doi: 10.1186/s12889-024-18008-9 (PMC10885545; doi:10.1186/s12889-024-18008-9)
Supplement: Supplementary file 1 — Supplementary Material 1 [file 12889_2024_18008_MOESM1_ESM.docx]

Supplementary Table1. Comparison of characteristics of study participants who were included and excluded in our study

|  | Included participants  (n=590)  [N/Mean±SD] | Excluded Participants  (n=885)  [N/Mean±SD] | | p-value* |
| --- | --- | --- | --- | --- |
| Gender of the child |  |  | |  |
| Boy | 307 | 467 | | 0.78 |
| Girl | 283 | 418 | |  |
| Birth weight (kg) | 3.2±0.4 | 3.2±0.4 | | 0.58 |
| Mother’s age (years) | 30.2±3.55 | 30.2±3.77 | | 0.66 |
| Mother’s Education |  |  | |  |
| <High school | 148 | 223 | | 0.18 |
| >=High school | 436 | 579 | |  |
| Parity |  |  | |  |
| 1 | 298 | 433 | | 0.06 |
| >1 | 242 | 334 | |  |
| Mother’s pre-pregnancy BMI (kg/m^3^) | 21.83±3.35 | 21.76±3.38 | | 0.76 |
| Mother’s allergy history |  |  | |  |
| No | 402 | 331 | | 0.53 |
| Yes | 188 | 140 | |  |
| Father’s allergy history |  |  | |  |
| No | 438 | 341 | | 0.49 |
| Yes | 151 | 129 | |  |
| Numbers may not match due to missing values. | | |  |  |

*p-value calculated using t-test for continuous variables and chi-square test for categorical variables.

Supplementary Table 2 Distribution of characteristics of study participants

| **Characteristics category** | **N [%]** |
| --- | --- |
| Mother’s education (years) |  |
| <=12 | 148 [25.08] |
| >12 | 436 [73.91] |
| Missing | 6 [1.01] |
| Pre-pregnancy BMI gp |  |
| <18.5 | 55 [9.32] |
| 18.5-22.9 | 276 [46.80] |
| >=23 | 138 [23.38] |
| Missing | 121 [20.50] |
| Parity |  |
| 1 | 298 [50.80] |
| >1 | 242 [40.71] |
| Missing | 50 [8.50] |
